# Structure–function analysis of the equine hepacivirus 5′ untranslated region highlights the conservation of translational mechanisms across the hepaciviruses

**DOI:** 10.1099/jgv.0.001316

**Published:** 2019-11-01

**Authors:** Joseph Lattimer, Hazel Stewart, Nicolas Locker, Andrew Tuplin, Nicola J. Stonehouse, Mark Harris

**Affiliations:** 1 School of Molecular and Cellular Biology, Faculty of Biological Sciences, and Astbury Centre for Structural Molecular Biology, University of Leeds, Leeds, LS2 9JT, UK; 2 Faculty of Health and Medical Sciences, School of Biosciences and Medicine, University of Surrey, Guildford, GU2 7XH, UK

**Keywords:** equine hepacivirus, 5′ untranslated region, selective 2′ hydroxyl acylation analysed by primer extension, internal ribosome entry site, translation

## Abstract

Equine hepacivirus (EHcV) (now also classified as hepacivirus A) is the closest genetic relative to hepatitis C virus (HCV) and is proposed to have diverged from HCV within the last 1000 years. The 5′ untranslated regions (UTRs) of both HCV and EHcV exhibit internal ribosome entry site (IRES) activity, allowing cap-independent translational initiation, yet only the HCV 5′UTR has been systematically analysed. Here, we report a detailed structural and functional analysis of the EHcV 5′UTR. The secondary structure was determined using selective 2′ hydroxyl acylation analysed by primer extension (SHAPE), revealing four stem–loops, termed SLI, SLIA, SLII and SLIII, by analogy to HCV. This guided a mutational analysis of the EHcV 5′UTR, allowing us to investigate the roles of the stem–loops in IRES function. This approach revealed that SLI was not required for EHcV IRES-mediated translation. Conversely, SLIII was essential, specifically SLIIIb, SLIIId and a GGG motif that is conserved across the *Hepaciviridae*. Further SHAPE analysis provided evidence that this GGG motif mediated interaction with the 40S ribosomal subunit, whilst a CUU sequence in the apical loop of SLIIIb mediated an interaction with eIF3. In addition, we showed that a microRNA122 target sequence located between SLIA and SLII mediated an enhancement of translation in the context of a subgenomic replicon. Taken together, these results highlight the conservation of hepaciviral translation mechanisms, despite divergent primary sequences.

## Introduction

As obligate intracellular parasites, viruses rely on the host cell machinery for translation. To avoid the complex and tightly regulated canonical initiation pathway, some viruses utilize internal ribosome entry sites (IRESs), which mediate direct recruitment of the ribosome in a 5′ cap-independent and 5′ end-independent fashion. Viral IRES elements have been classified into six types, depending upon their structure and requirement for host cell factors, termed picornavirus type I–V IRESs and intergenic region IRESs [1–7].

Type IV IRESs are also known as HCV-like IRESs, as the 5′ untranslated region (5′UTR) of hepatitis C virus (HCV), contains a series of RNA structures that cooperatively direct both ribosome assembly and initiation of cap-independent translation of the viral polyprotein. The 5′UTR of equine hepacivirus (EHcV, previously termed non-primate hepacivirus and now also classified as hepacivirus A), the most closely related virus to HCV, has also been described to function as an IRES [8] and constitutes another type IV IRES. However, whilst HCV is a worldwide health concern causing significant liver pathology in chronically infected people, EHcV appears to possess limited pathogenic potential and is cleared in the majority of cases in its natural host, the horse [9–12]. Investigating the replication mechanisms of this putative HCV model is important to identify which are the causative elements underlying these divergent pathologies.

However, to date, an infectious clone able to replicate in tissue culture is not yet available, limiting comparative studies.

**Fig. 1:**
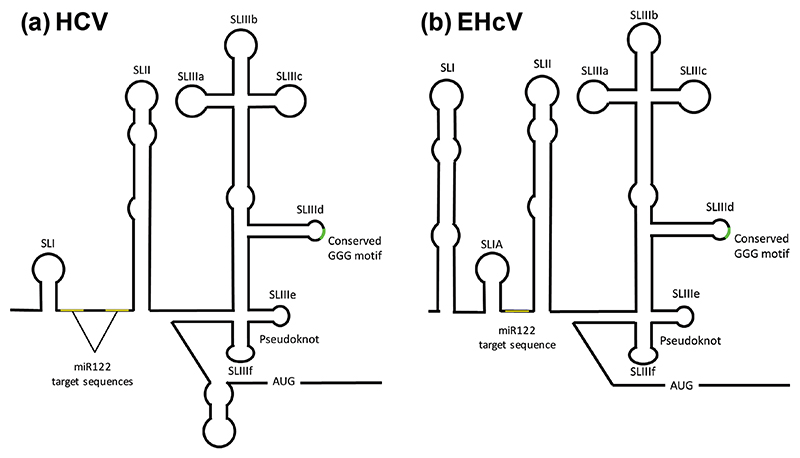
Structure of the HCV and EHcV 5′UTRs. (a) Experimentally determined structure of the HCV 5′UTR showing the location of the miR122 target sequences, stem–loops SLI–IV, the pseudoknot and polyprotein AUG. (b) Predicted structure of the EHcV 5′UTR.

The HCV 5′UTR is 341 nucleotides in length and comprises 4 stem–loops (SL) – SLI–IV – and a pseudoknot (Fig. 1a). SLI only functions in replication, playing no role in translation. The remainder of the 5′UTR comprises the IRES, however, SLIII and SLIV have been demonstrated to exhibit IRES activity in the absence of SLII [13, 14]. The HCV IRES directly recruits the ribosomal 40S subunit and has been reported to require only a minimal subset of initiation factors: eIF3, eIF5, eIF5B and the eIF2-GTP-^Met^tRNA ternary complex (reviewed in [15]). Hence, there is no requirement for ribosome scanning, with SLIIId and the pseudoknot facilitating loading of the 40S ribosomal subunit directly on the AUG initiation codon [16]. The initial 42 nucleotides of the coding region also contribute to efficient translation [17].

Two critical interactions are required for ribosome recruitment by the HCV IRES. Firstly, 40S recruitment is mediated through a direct interaction between a GGG motif in the apical loop of SLIIId and the _1116_CCC_1118_ motif in the 18S ribosomal RNA [18–20]. Mutation of this motif reduces the affinity of the IRES for the 40S subunit and severely impairs translation [21, 22]. Secondly, eIF2 is recruited to the 40S subunit via an RNA-dependent interaction with eIF3. The eIF2–eIF3–40S interaction is dependent upon specific interactions between SLIIIb (the apical loop and a mismatched bulge within the stem [23, 24]) and the ribosome-binding face of eIF3. Consequently, mutations in these regions of SLIIIb also inhibit IRES activity [21].

We previously described the IRES function of the EHcV 5′UTR [8], however, to date, there is only limited information available regarding the structure and function of this type IV IRES [8, 25–27]. The EHcV 5′UTR exhibits 66% nucleotide identity with its HCV counterpart and a minimum free energy analysis of the 5′UTR predicted a large 5′ SLI followed by three SLs (SLIa, SLII and SLIII), analogous to HCV SLI–III, and a pseudoknot (Fig. 1b). The major differences between EHcV and HCV were the presence of the large 5′ SLI and a lack of SLIV. Functional analysis of the EHcV 5′UTR demonstrated that IRES activity was not affected by a deletion of SLI and the role of this structure in the viral life cycle has yet to be elucidated [8]. The EHcV 5′UTR was also enhanced in the presence of the cognate 3′UTR and the liver-specific microRNA122 (miR122) [27], despite the fact that it only possesses one target sequence in comparison to the two within the HCV 5′UTR [28–30]. Importantly, two recent studies [27, 31] have shown that the EHcV 5′ SLI can function to support HCV genome replication when substituted for the smaller HCV SLI, which is suggestive of a commonality of function.

The 5′UTRs of these two closely related viruses therefore exhibit an unexpected combination of highly conserved regions and significant structural differences, and it cannot therefore be assumed that the mechanism of translation initiation is conserved between them. For example, although SLI is not required for EHcV IRES function [8] it may alter the interaction of other RNA domains with individual eIFs. To address these questions, we carried out a structural and functional analysis of the EHcV 5′UTR. This report describes the experimental confirmation of the secondary structure using selective 2′ hydroxyl acylation analysed by primer extension (SHAPE); this structure informed a mutational analysis to investigate how structure related to IRES function. Footprinting analysis was utilized to investigate EHcV 5′UTR interactions with the host cell translational machinery, specifically eIF3 and the 40S ribosomal subunit.

## Results

### Experimental determination of the EHcV 5′uTR secondary structure

We [8] and others [27] have previously demonstrated that the 5′UTR of EHcV functions as an IRES and is able to efficiently drive translation of bicistronic reporter constructs, monocistronic expression constructs and an SGR. Although the RNA secondary structure of the 5′UTR has been predicted, it has not been experimentally confirmed. To address this, SHAPE experiments were therefore performed upon the 5′UTR that we had previously derived by RT-PCR from the serum of a persistently infected horse, and importantly had been shown to be competent for cap-independent initiation of translation [8]. The values obtained from SHAPE were used in the prediction of RNA secondary structure as pseudo-free energy constraints in the prediction software RNAstructure. To ensure that the information obtained from this analysis was physiologically relevant we used *in vitro* transcription to generate full-length EHcV SGR RNA as a template for the SHAPE reactions. This would ensure that any effect of long distance RNA–RNA interactions (e.g. between UTRs) on the structure of the 5′UTR would be preserved. A pseudoknot is predicted to form in the EHcV 5′UTR; such tertiary structures will disrupt the structure predictions in their immediate vicinity. For this reason, SLIIIe, SLIIIf and the pseudoknot were manually modelled and the SHAPE reactivities were subsequently mapped on to the structure. We are confident that this approach provides an accurate representation of RNA secondary structure in this region, especially when the sequence similarity to HCV is taken into account.

**Fig. 2:**
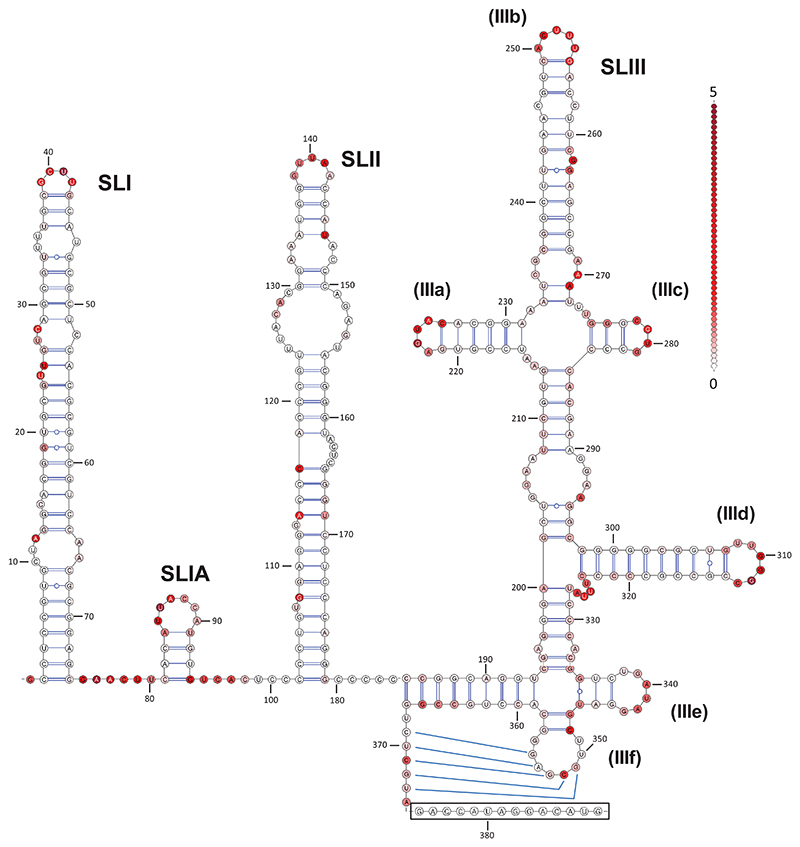
Structure of the EHcV 5′UTR informed by thermodynamic predictions and experimentally determined SHAPE constraints. SHAPE was performed on the WT EHcV 5′UTR in the context of the full-length *in vitro*-transcribed EHcV SGR RNA. SHAPE was conducted to *n*=2 and an average value was taken from these data. SHAPE reactivity values were used as a pseudo-free energy constraint in the RNAstructure program. The pseudoknot region was manually modelled based on conservation with HCV and previously described data [25, 26]. SHAPE reactivities are represented on a colour scale from white (low reactivity – predicted paired) to red (high reactivity – predicted unpaired). A representative scale denoting reactivity increments of 0.1 is displayed. Number labelling is in accordance with the nucleotide position in the NZPI consensus sequence. The identities of the various stem–loops (SLs) and individual loops within SLIII (a–f) are indicated. The box shows residues whose reactivity could not be determined; this includes the initiation codon for the EHcV polyprotein (residues 386–388).

The resulting experimental determination of the EHcV 5′UTR RNA secondary structure is presented in Fig. 2 and represented graphically in Fig. S1 (available in the online version of this article). There are only minor differences between this structure and those predicted previously from the sequences of other EHcV isolates [25, 26]; the majority of these discrepancies concern the unpaired nucleotides within SLII and the size of the terminal loop of SLIIIb – the latter may be due to sequence variation between our isolate and other published EHcV clones (see Table 1 below). The experimental data generally agree well with the predicted structure of the NZPI isolate, showing that the EHcV 5′UTR adopts a modular structure formed by the three major stem–loops SLI, SLII and SLIII, together with the short SLIA. The overall architecture of SLIII is as predicted and shows a high level of structural homology with HCV. However, unlike HCV, the EHcV lacks the final stem–loop (SLIV) and the polyprotein AUG is located much closer to the 5′UTR. HCV-like IRES structures lacking SLIV have been documented previously, but not in such closely related viruses [32–34]. Due to experimental limitations, reactivity values were not available for nucleotides 375–388; the reasons for this are unclear, but might, for example, result from reverse transcriptase stuttering. To the best of our knowledge, this represents the first experimentally confirmed model of the EHcV 5′UTR.

### SLIII is essential to EHcV IRES activity

**Fig. 3:**
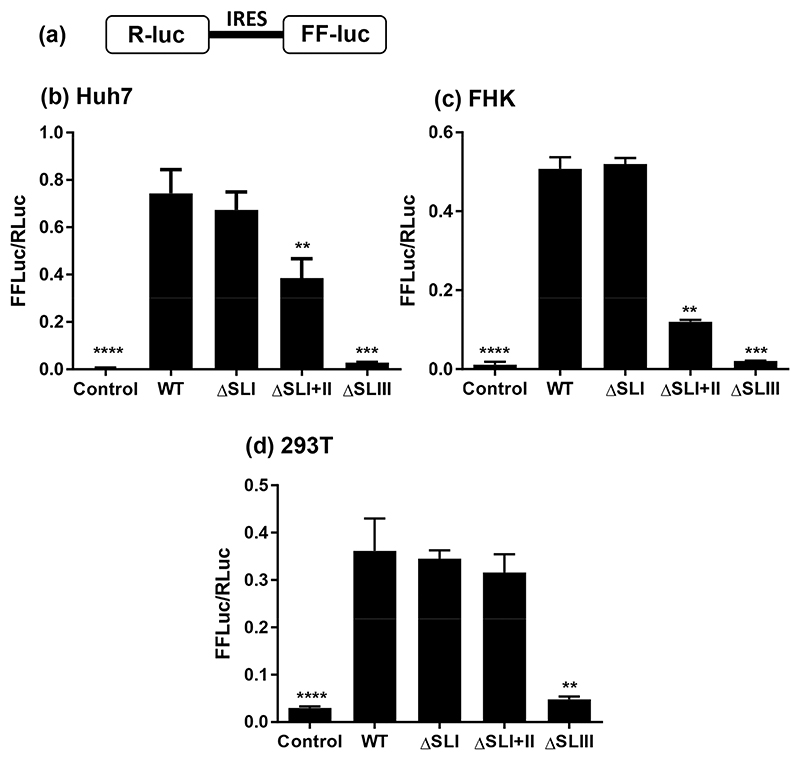
IRES activity of 5′UTR deletion mutants in the context of a bicistronic plasmid construct. (a) Structure of the bicistronic vector, pRF. (b–d) The indicated cell lines were transfected with DNA plasmids using PEI and harvested at 24 h p.t. The ratio of firefly (FF) luciferase to *Renilla* (R) luciferase is presented. Significant differences from wild-type (WT) denoted by ** (*P*<0.01), *** (*P*<0.001) or **** (*P*<0.0001).

In order to investigate how the structure of the EHcV 5′UTR related to its function as an IRES, the following nucleotides (inclusive) were deleted from the IRES to create a series of mutants (numbering based upon Fig. 2): ΔSLI: 1–71; ΔSLI+II: 1–177; ΔSLIII: 193–360; ΔSLIIIb: 239–268; ΔSLIIId: 299–322. The wild-type (WT) and deletion mutant EHcV 5′UTR sequences were introduced into a bicistronic vector (pRF), containing both the *Renilla* (R) and firefly (FF) luciferase ORFs (kindly provided by Kensuka Hirasawa [35]). Sequences were cloned between the two luciferase ORFs, such that the initial 10 residues of the EHcV predicted polyprotein were in-frame with that of FF luciferase and expression of the latter was under the translational control of the inserted EHcV 5′UTR sequence. An IRES-free control vector (pRZF) was also used to assess background FF expression (control). Plasmids were transfected into Huh7, FHK and 293T cells and cell lysates were harvested at 24 h post-transfection (p.t.) for the determination of both RL and FF luciferase activity. The ratio of the two gives a measure of IRES activity and is presented in Fig. 3. As we previously reported [8], the deletion of SLI (ΔSLI) did not exert any significant effect on translation from the EHcV IRES, indicating that SLI is not involved in EHcV translation. Deletion of both SLI and SLII (ΔSLI+II) caused a 50% reduction in translation compared to WT in Huh7 cells and a 75% reduction in FHK cells. However, this deletion had no apparent phenotype in 293T cells. Deletion of SLIII caused a complete ablation of translation in all cell types, with luciferase levels equivalent to pRZF transfection.

**Fig. 4:**
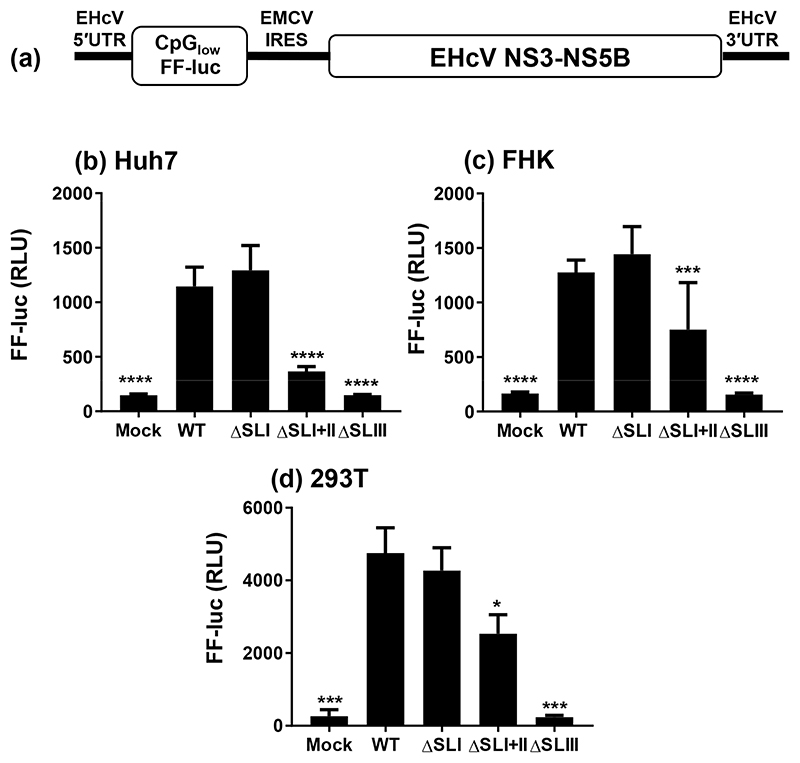
IRES activity of 5′UTR deletions in the context of the EHcV SGR. (a) Structure of the EHcV SGR. (b–d) The indicated cell lines were electroporated with EHcV SGR RNA, either WT or the indicated SL deletions. Cells were harvested at 6 h p.t. and assayed for FF luciferase activity. Significant differences from WT denoted by: * (*P*<0.05), *** (*P*<0.001) or **** (*P*<0.0001).

Whilst the use of bicistronic vectors is an accepted technique for measuring IRES function, we considered that in the case of the EHcV 5′UTR the internal location of the IRES might not reflect the physiological situation, i.e. where the IRES is located at the 5′ end of an RNA molecule. We therefore also cloned the WT EHcV 5′UTR and the deletions into the EHcV SGR, pNZCI-luc, replacing the WT 5′UTR in this construct. pNZCI-luc is an adaptation of pNZPI-SGR [27], in which the neomycin phosphotransferase gene was replaced by a derivative of the FF luciferase gene engineered to minimize the occurrence of either CpG or UpA dinucleotides (low CpG/UpA-luc), as described previously [36]. RNA was transcribed *in vitro* and Huh7, FHK and 293T cells were electroporated with RNA, harvested at 6 h p.t. and assayed for FF luciferase activity (Fig. 4). The results largely reflected those seen with the bicistronic vector – ΔSLI had no effect, whereas ΔSLI+II caused a significant reduction in translation compared to WT; in Huh7 cells approximately 75% compared to FHK and 293T cells (~50%). ΔSLIII reduced FF luciferase levels to those of mock transfection. Taken together, these data indicate that SLIII and the pseudoknot are necessary and sufficient for EHcV IRES function.

**Fig. 5:**
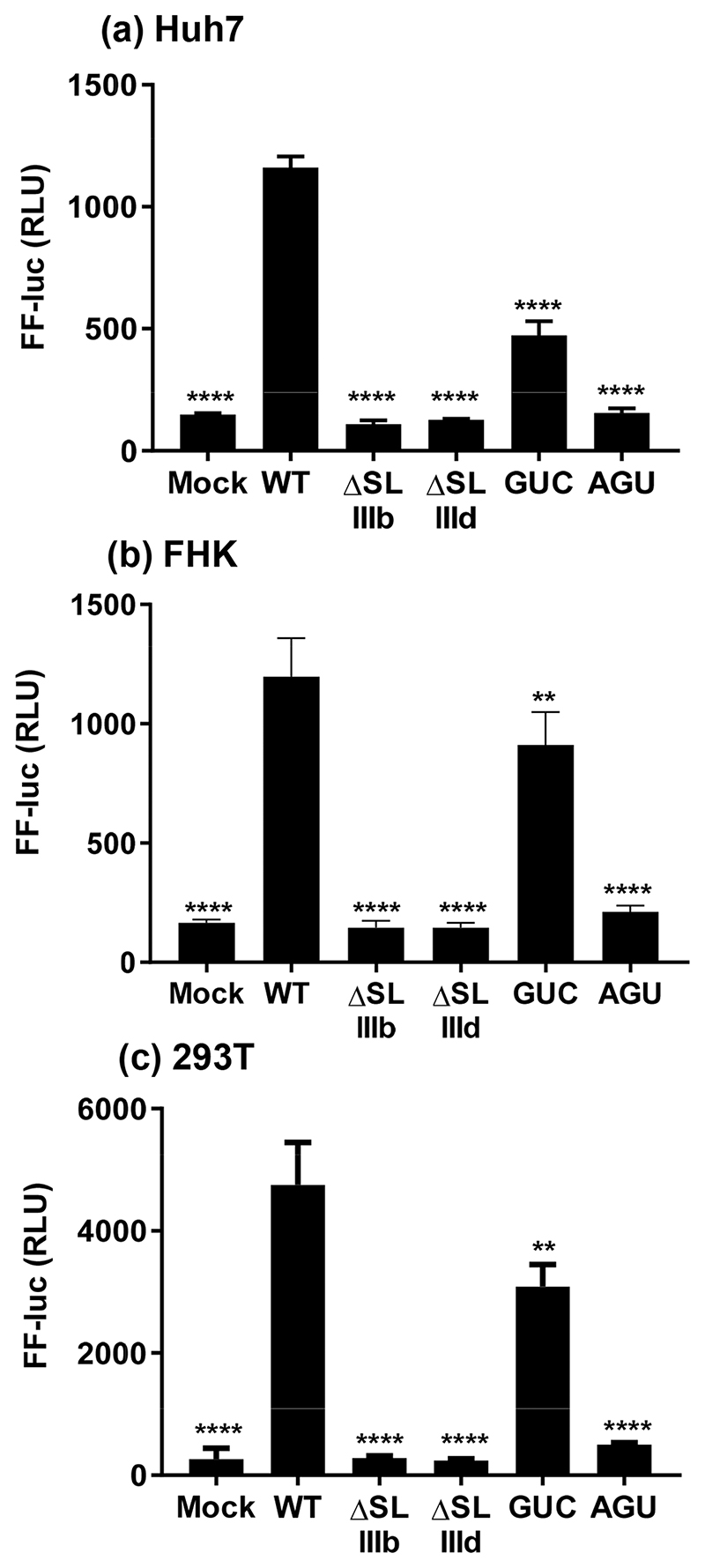
IRES activity of 5′UTR deletions and substitutions in the context of the EHcV SGR. The indicated cell lines were electroporated with EHcV SGR RNA, either WT or the indicated SL deletions/substitutions. Cells were harvested at 6 h p.t. and assayed for FF luciferase activity. Significant differences from WT are denoted by: ** (*P*<0.01) or **** (*P*<0.0001).

In the HCV 5′UTR key roles have been demonstrated for the two loops (SLIIIb and SLIIId) in the initiation of translation via interactions with eIF3 and the 40S ribosome, respectively [21–23, 37]. To test whether these structures were also required for EHcV IRES function we generated deletions of each loop (ΔSLIIIb and ΔSLIIId) in the context of the EHcV SGR, pNZCI-luc. Deletion of either loop completely abrogated FF luciferase translation, indicating that these structures were likely functioning in a similar fashion to HCV (Fig. 5). As deletions could have led to larger scale changes in IRES structure, we introduced specific substitution mutations into SLIIIb (_251_CUU_253_ to _251_**G**U**C**_253_, referred to as GUC) and SLIIId (_310_GGG_312_ to _310_**A**G**U**_312_, referred to as AGU). This latter mutation has previously been found to ablate both translation and 40S ribosomal subunit interactions within the HCV IRES. The GUC substitution exhibited a significant impairment of translation, to ~40% of WT in the Huh7 cells and ~75% in FHK/293T cells. However, unlike the ΔSLIIIb deletion, GUC did not cause a complete ablation of translation, indicating that the presence of an extended SLIIIb helix is required for EHcV translation, regardless of the apical loop sequence. In contrast, the AGU substitution displayed an almost complete ablation of translation in all three cell types, confirming that the sequence of the SLIIId apical loop is a key determinant of EHcV IRES activity, consistent with the results obtained for HCV [37].

### SHAPE footprinting reveals that EHcV SLIII interacts with eIF3 and the 40S ribosomal subunit

The functional analysis suggested that, as observed for HCV, the GUC and AGU substitutions could be disrupting SLIIIb and SLIIId interactions with eIF3 and the 40S ribosomal subunit. To test this hypothesis, SHAPE footprinting analysis of SLIII was conducted in the presence of either purified human eIF3 or 40S ribosomal subunit. In conventional SHAPE the reactivity of any given base is dependent upon the RNA backbone conformation and the associated orientation or accessibility of the 2′OH groups. This can be altered by interactions with ligands such as proteins [38]. The addition of purified protein before NMIA treatment will therefore stabilize a particular RNA conformation and may exert a protective effect, precluding subsequent NMIA binding. This may shift individual protein-binding nucleotides from a reactive to unreactive state.

**Fig. 6:**
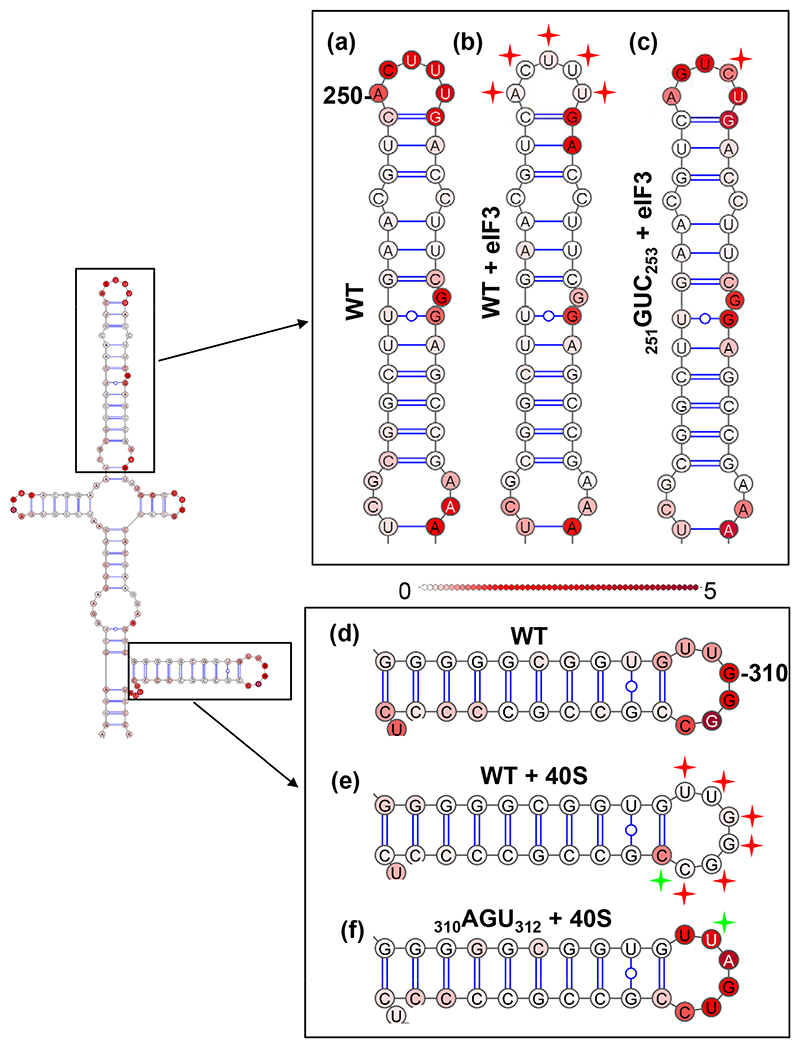
Mutations within the apical loops of SLIIIb and SLIIId ablate interactions with eIF3 and the 40S ribosomal subunit, respectively. The indicated regions of SHAPE footprinting analyses are expanded. (a–c) SLIIIb: WT or _251_**G**U**C**_253_ 5′UTR in the absence (a) or presence (b, c) of eIF3. (d-f) SLIIId: WT or _310_**A**G**U**_312_ 5′UTR in the absence (d) or presence (e, f) of the 40S ribosomal subunit. SHAPE footprinting was conducted to *n*=3 and a two-tailed Student’s *t*-test was performed for every nucleotide in (b) and (c) in comparison to (a), and (e) and (f) in comparison to (d). Red stars indicate nucleotides that demonstrated a significant decrease in SHAPE reactivity upon the addition of either eIF3 or the 40S ribosomal subunit compared to WT, no protein. Green stars represent nucleotides that exhibited a significant increase.

The ability of SLIIIb and SLIIId to interact with eIF3 and the 40S ribosomal subunit was investigated using SHAPE foot-printing. WT EHcV 5′UTR RNA was transcribed *in vitro* and subjected to SHAPE footprinting analysis in the absence (Fig. 6a, d) or presence of purified eIF3 (Fig. 6b) or 40S ribosomal subunit (300 nM) (Fig. 6e) This value was chosen from previous studies as being at, or above, the expected Kd for HCV-like IRES elements [39]. Numerical SHAPE data for these experiments are presented in Table S1 and represented graphically in Figs S2 and S3. The purity of the 40S ribosomal subunit and eIF3 preparations is presented in Fig S4.

The apical loop of WT SLIIIb (_250_ACUUU_254_) was highly NMIA-reactive when analysed in the absence of protein (Fig. 6a). However, upon the addition of eIF3, NMIA reactivity was significantly reduced across all five bases in the apical loop (Fig. 6b). No other statistically significant changes in NMIA reactivity were observed in SLIIIb in the footprinting assay, although the unpaired G_262_ in the bulge exhibited a non-significant reactivity decrease. These data indicate that eIF3 was specifically interacting with the apical loop of EHcV SLIIIb.

Similarly, the apical loop of SLIIId (_307_GUUGGGCC_314_) was highly NMIA-reactive in the absence of interacting partners (Fig. 6d). However, upon the addition of the 40S ribosomal subunit, NMIA reactivity was significantly reduced across all seven bases of the apical loop (Fig. 6e). These data indicate that the 40S ribosomal subunit was specifically interacting with the apical loop of EHcV SLIIId.

SHAPE was also conducted across the SLIII of both the GUC and AGU substitutions in the absence of protein. No significant differences were observed between these data and those obtained for the WT NZCI (data not shown), consistent with the conclusion that the changes in reactivity described above are mediated by protective protein interactions and do not reflect altered RNA structure.

The addition of eIF3 and the 40S ribosomal subunit significantly altered the NMIA reactivity of bases in the apical loops of SLIIIb and SLIIId, respectively, which was indicative of protein–RNA or RNA–RNA interactions (Fig. 6b and e). We therefore hypothesized that the reduced translation of the GUC and AGU substitutions (Fig. 5) was caused by disruption of these interactions. To test this hypothesis, these mutants were subject to SHAPE footprinting assays as described for the WT EHcV 5′UTR.

Unlike the WT EHcV 5′UTR, the GUC substitution exhibited very little change in NMIA reactivity across the apical loop of SLIIIb in the presence of eIF3 compared to the protein-free WT (Fig. 6c). Only _253_U/C exhibited a significant change (reactivity from 2.27 decreasing to 0.77), however, as the SHAPE reactivity at this residue is greater than 0.7, it is still considered to be highly reactive [38, 40]. The unpaired _262_G in the stem also regained a similar reactivity level as observed in the protein-free WT control. These data confirm that mutations within the apical loop of SLIIIb disrupt RNA–eIF3 interactions in the EHcV IRES.

Similarly, upon the addition of the 40S ribosomal subunit, the AGU substitution exhibited no significant decreases in NMIA reactivity across all bases of the apical loop (Fig. 6f). The only change in NMIA reactivity was observed at _309_U; this was a significant increase compared to the WT 40S ribosomal subunit-free control. Taken together, these data confirm that the 40S–RNA interaction is mediated through the apical loop of SLIIId and mutations in this region prevent this interaction.

### The miR122 target sequence influences EHcV IRES-mediated translation

**Fig. 7:**
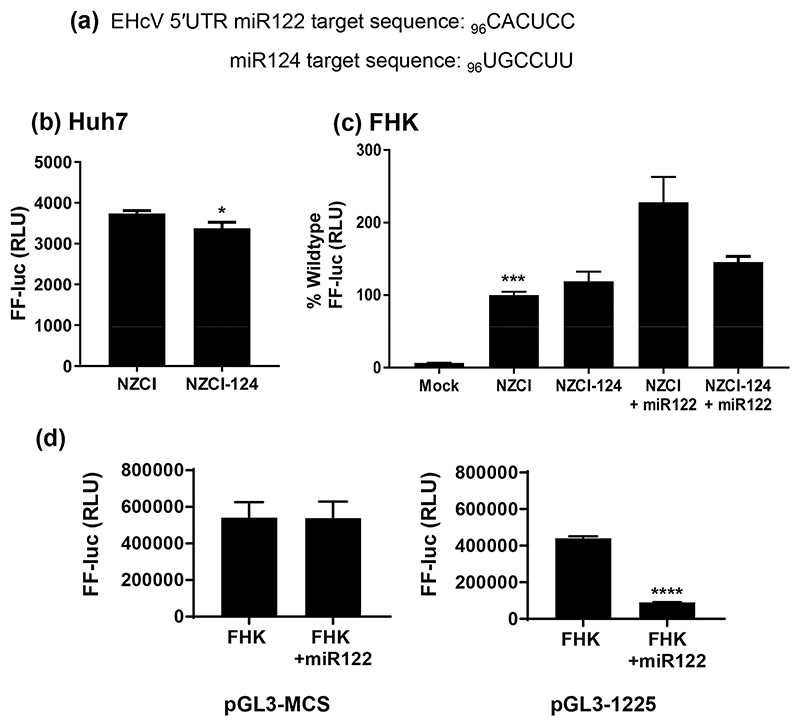
Functional analysis of the miR122 target sequence in the EHcV 5′UTR. (a) Sequence of the miR122 and miR124 target sequences (complementary to the seed sites). (b, c) The indicated cell lines were electroporated with EHcV SGR RNA, either WT or the miR124 target sequence substitution. Cells were harvested at 6 h p.t. and assayed for FF luciferase activity. Significant differences from WT (NZCI) are denoted by: * (*P*<0.05) or *** (*P*<0.001). (d) A control FF luciferase reporter pGL3-MCS or the miR122-responsive pGL3-1225 was transfected into either parental FHK or FHK/miR122 cells, and assayed at 24 h p.t. for FF luciferase activity. Significant differences from parental FHK cells are denoted by: **** (*P*<0.0001).

In the HCV 5′UTR there are two sequences (5′-CACUCC) located between SLI and SLII that are complementary to the seed site (bases 2–8) of miR122 and mediate binding to this microRNA (Fig. 1a). Expression of miR122 is restricted to the liver *in vivo* and has been shown to be required for HCV replication and to modulate HCV translation [41]. In contrast, the EHcV 5′UTR only contains one miR122 target sequence, located between SLIA and SLII (Fig. 1b). To assess the potential role of miR122 in EHcV IRES function, the miR122 target sequence was mutated to the corresponding miR124 target sequence (UGCCUU) (Fig. 7a) in the context of the EHcV SGR, pNZCI-luc. Surprisingly, when these RNAs were transfected into Huh7 cells a modest yet significant reduction in FF luciferase expression was observed for the miR124 derivative (Fig. 7b), suggesting that miR122 binding was not absolutely required for EHcV translation. We proceeded to test this in a different cell type, FHK, which are kidney cells and thus would not be expected to express miR122. As a control for this experiment, FHK cells were also transduced with a lenti-virus to express miR122. FHK and FHK-miR122 cells were subsequently transfected with RNA for either WT NZCI-luc or the miR124 derivative and FF luciferase expression was compared (Fig. 7c). This analysis indicated that the exogenous expression of miR122 significantly stimulated FF luciferase translation from the WT EHcV 5′UTR but had no effect on the miR124 derivative. To confirm the functionality of the lentivirus-delivered miR122 in FHK cells, we transfected the parental and FHK-miR122 cells with a control vector (pGL3-MCS) or a vector containing an miR122 target sequence such that luciferase expression was inhibited by miR122 binding (pGL3-1225; a kind gift from Dr Catherine Jopling, University of Nottingham). Luciferase levels were similar in FHK cells transfected with either the miR122-responsive construct or the control, indicating a lack of endogenous expression of miR122. However, in the FHK-miR122 cells luciferase levels from the miR122-responsive construct were significantly lower than the control. This analysis confirmed that FHK did not endogenously express miR122, but that the lentivirus-delivered miR122 was functional (Fig. 7d). These data confirm that EHcV IRES activity can be enhanced by exogenous expression of miR122, and that enhancement is mediated by the miR122 target sequence located between SLIA and SLII.

## Discussion

### SLI and SLII are dispensable for EHcV IRES activity

This study provides the first experimental confirmation of the secondary structures within the EHcV 5′UTR (Fig. 2) and delineates the essential IRES as consisting of SLIII and the adjacent pseudoknot, whilst the preceding SLI, SLIA and SLII are not required for minimal IRES activity (Figs 3 and 4). However, whilst the deletion of SLI alone had no effect on translation efficiency, the absence of SLI and SLII together caused a significant impairment, indicating that SLII may contribute to IRES function indirectly through ribosomal contacts. This is analogous to the HCV IRES, where truncation and substitution mutants of SLII led to a similar level of translation reduction (15–25% of WT levels) [42]. It has been suggested that SLII facilitates 80S ribosome assembly by promoting eIF5-induced GTP hydrolysis and eIF2/GDP release [43], but nevertheless remains dispensable [44–47]. During the preparation of this manuscript a similar study analysing the function of the EHcV 5′UTR in translational initiation [48] was published. The two studies are in partial agreement – for example, both demonstrate the absolute requirement for SLIII in IRES activity (in particular loops SLIIIb and SLIIId) – but there are some discrepancies that merit discussion. Notably, Tanaka *et al*. showed that deletion of SLII abolished IRES activity, and deletion of SLI reduced activity by ~50% [48]. The precise locations used by Tanaka *et al*. to define the SLI and SLII deletions are not reported, so it is possible that subtle differences might have profound functional effects. In addition, there are several differences in the sequences of the 5′UTRs used in the two studies, particularly in the region corresponding to SLIA (termed I′ by Tanaka *et al*.), in SLII and the apical loop SLIIIb (Table 1). The fact that such a large stable structure as SLI (_2_C-G_74_) is present at the extreme 5′ terminus of the EHcV genome, and yet clearly plays no role in translation, is intriguing. The HCV SLI functions in replication, whilst SLII–IV and the pseudoknot contribute to IRES activity [8, 15, 27, 49]; it is therefore not unreasonable to predict a role for EHcV SLI in RNA replication. Consistent with this, replacement of the HCV SLI with the EHcV SLI resulted in a 10-fold increase in HCV sub-genomic replicon replication, as judged by a colony formation assay, but only a modest increase in HCV IRES function [48]. The latter observation suggests that the EHcV SLI might function in translation via long-range interactions with the cognate coding region or 3′UTR. In this context, it is noteworthy that the EHcV 3′ UTR differs from its HCV counterpart in possessing a long (~100 nucleotide) poly-U tract. In addition, the presence of the EHcV 3′UTR stimulated translation from the 5′UTR [27], consistent with the existence of long-range interactions between the two UTRs.

It is notable that many of the unpaired ‘bulge’ nucleotides within the SLI helix appeared protected from NMIA reactivity during SHAPE analysis, which cannot be due to ligand-mediated protection. This may be due to the relatively slow reaction rate of NMIA [50]; the NMIA reactivity would reflect an ‘averaged’ value if the RNA were switching rapidly between transient conformations [51]. The consistently high reactivity of the SLI apical loop suggests that any conformational changes of this nature do not involve pairing of these nucleotides at any point. A similar situation may be occurring across SLII; the unpaired nucleotides within this structure do not appear to be highly reactive, for example, compared to the terminal loop. A situation can be envisaged where SLI and SLII represent a structurally flexible subdomain upstream of the essential IRES, sequentially forming a series of conformations as they interact with specific host or viral factors to regulate each stage of the viral replication cycle. This is in contrast to the highly conserved and stable structures of SLIII and the pseudoknot, which form a prototypic type IV IRES and are absolutely essential for viral translation. The observation that ΔSLI+II retains almost full activity in 293T cells (Fig. 3d) may be pertinent here, as it alludes to cell type-specific interactions with the minimal IRES (SLIII) to initiate translation; for example, there may be differing levels or activities of IRES trans-acting factors (ITAFs) in different cells.

### eIF3 interacts with the apical loop of SLIIIb during EHcV IRES-mediated translation.

Whilst the deletion of SLIIIb abrogated the translational initiation activity of the EHcV IRES, the _251_CUU_253_ -to-_251_ **G**U**C**_253_ mutation within the apical loop (GUC) merely reduced activity to 40–70% of WT, indicating that the tertiary structures created by this helix are essential regardless of the apical loop sequence. Supporting this is evidence that the SLIIIabc triple-helix junction has been identified as an important determinant of HCV IRES-eIF3 interactions that modulates translation efficiency [52]. Although our data suggest a role for the SLIIIabc junction in EHcV translation, eIF3 footprinting did not indicate any site-specific interactions with either SLIIIa or SLIIIc, only the apical loop of SLIIIb. However, it is probable that additional minor interactions contribute to the stability of the RNA–eIF3 complex and, as such, it may retain a low-affinity interaction in the cell-based assays, thereby allowing low levels of translation despite the GUC substitution. This is supported by previous reports that a deletion of the apical portion of HCV SLIIIb resulted in 34% translation efficiency of WT, similar to that observed here for the GUC substitution [23]. Of note, both the sequence and the length of SLIIIb appear to be quite variable between different isolates of EHcV, whereas the flanking sequences are conserved (Table 1).

Intriguingly, the HCV study also identified that _214_AAU_216_, which resides within a mismatched loop in the HCV SLIIIb, interacted with eIF3. Although this loop is not conserved with EHcV SLIIIb, we observed a non-significant decrease in NMIA reactivity at the mismatched G within SLIIIb, which was reliant upon eIF3 protection. This suggests that RNA–eIF3 interactions extend beyond the main site of the apical loop, and similarly extensive interactions may be conserved across the viral species despite alterations in primary sequence. Conversely, unlike studies conducted on eIF3–HCV 5′UTR interactions, no significant reduction in NMIA reactivity was observed across the other SLIII apical loops. The apical loops of SLIIIa and SLIIIc are completely conserved between HCV and EHcV, suggesting that they share a conserved function and mutation of these SLs in HCV reduced translation to <10% of WT. The HCV/EHcV similarities in translation initiation, and specifically those interactions involving RNA subdomains and eIF3, cannot therefore be said to be identical, although they exhibit similar features. The requirement for SLIIIb-eIF3-specific interaction indicates that EHcV may enhance viral translation by preventing the accumulation of 43S complexes and promoting the availability of 40S subunits, in a similar manner to that seen in HCV and the related pestiviruses [23, 53].

### The 40S ribosomal subunit interacts with _310_GGG_312_ during IRES-mediated translation

The deletion of SLIIId completely ablated translation from the EHcV IRES; our results indicate this was due to the absence of the GGG motif within the apical loop. The analogous motif in HCV interacts with _1116_CCC_1118_ of the 18S ribosomal RNA component of the 40S ribosomal subunit, leading to a structural rearrangement of the 40S : IRES complex and positioning the 40S subunit at the initiation codon [19, 20, 37]; accordingly, this exerts a protective effect on _266_GGG_268_ in the HCV apical loop [37]. SHAPE footprinting analysis confirmed the conservation of this site-specific interaction: the 40S ribosomal subunit had a protective effect on the apical loop of EHcV SLIIId, with all bases in the apical loop exhibiting a significant reduction in NMIA reactivity in this experiment. No bases exhibited a loss of NMIA reactivity upon the addition of the 40S subunit to the AGU mutant of NZCI. The structural, sequence and functional conservation of these bases between EHcV and HCV is strongly indicative that _310_GGG_312_ of EHcV SLIIId interacts with _1116_CCC_1118_ of the 18S RNA component of the 40S ribosomal subunit.

The conserved GGG motif could be the key factor in understanding the clearly essential nature of SLIIId in translation from the EHcV IRES. The conservation of the GGG motif in the apical loop extends across both the *Hepaciviridae* and the related *Pestiviridae* [22]. It is likely, therefore, that the mechanism of translational initiation is conserved between the EHcV and HCV IRES structures. If this is the case, then the SLIIId deletion within NZCI would disrupt the IRES : 40S interaction, therefore preventing ribosome recruitment and the subsequent formation of a translation complex.

### miR122-mediated enhancement of EHcV IRES activity is dependent on a miR122 target sequence.

miR122 is a liver-specific microRNA that is essential to HCV RNA replication; however, it has also been demonstrated to play a role in translation and RNA stability [30, 41, 54]. Whilst HCV contains two miR122 target sequences within its 5′UTR, EHcV contains only one, located directly upstream of SLII. Notably, this is similar to the recently described bovine hepacivirus IRES, which also only possesses one target sequence immediately upstream of SLII and exhibits miR122-enhanced translation [55].

Our data demonstrate that mutation of the miR122 target to the corresponding miR124 target did not disrupt IRES-mediated translation in Huh7 cells (Fig. 7b). This is in agreement with previous studies [27, 48] showing that in Huh7 cells neither sequestration of miR122 with a locked nucleic acid, nor ectopic expression of miR122, had any effect on translation from the EHcV IRES. In contrast, it has been previously demonstrated that the ectopic expression of miR122 in equine fibroblasts (E. Derm cells), in which miR122 is not endogenously expressed, resulted in a modest upregulation of translation from the EHcV 5′UTR IRES [27]. In this report we have both confirmed the enhancement of translation mediated by ectopic expression of miR122 in foetal horse kidney (FHK) cells, and shown that this requires the predicted target sequence between SLIA and SLII. Thus it appears the effect of miR122 on EHcV translation is cell type-dependent; one possibility is that this effect is indirect and miR122 is regulating expression of host cell proteins required for IRES activity. In the case of HCV it is clear that miR122 functions via multiple mechanisms to enhance both translation and genome replication, for example by protecting from *XrnI* exonuclease and modifying the structure of the 5′UTR [54, 56–58], and it seems likely that this is also the case for EHcV.

### Specific features of translation initiation are conserved across the *Hepaciviridae*

In this study a complementary functional and structural analysis of the EHcV 5′UTR was undertaken that conclusively demonstrated that SLI is not involved in IRES-mediated translation, SLII is not essential but enhances translation and SLIII is absolutely necessary. The apical loop of SLIIIb interacts with eIF3 and, whilst this site-specific interaction is not absolutely required for IRES function, the presence of SLIIIb (and the tertiary structures to which it contributes) are essential for IRES function. SLIIId and the sequence within its apical loop are both required for IRES activity, due to interactions between the conserved GGG motif and the 40S ribosomal subunit. Based upon these data, we propose that specific features of translation initiation are conserved between EHcV and HCV. Future investigations should examine whether such conservation extends to other recently identified hepaciviruses that possess distinctly divergent 5′ structural features.

## Methods

### Cell culture

Human hepatocellular carcinoma (Huh7) [59], foetal horse kidney (FHK) [60] and 293T human embryonic kidney cells were maintained in Dulbecco’s modified Eagle’s medium (DMEM; Sigma) supplemented with 10% foetal bovine serum, 100 IU ml^−1^ penicillin, 100 μg ml^−1^ streptomycin and 1 mM non-essential amino acids in a humidified incubator at 37 °C with 5% CO_2_.

### DNA constructs

The EHcV subgenomic replicon (SGR), pNZCI-luc, was adapted from the previously reported pNZPI-SGR (GenBank accession no. KP325401) [27], which contained a neomycin-resistance reporter gene and the non-structural protein-coding region of EHcV, separated by an encephalomyocarditis virus IRES, flanked by the 5′ and 3′UTRs. To create pNZCI-luc, the neomycin phosphotransferase gene was replaced with a FF luciferase gene engineered to minimize the occurrence of CpG/UpA dinucleotides [36]. In addition, the 5′UTR was replaced with a sequence that was previously derived from a persistently infected horse [8].

### DNA transfection

Plasmids were transfected using polyethylenimine (PEI). Briefly, DNA (2 μg) was diluted in 100 μl Optimem (Sigma), mixed with 10 μl 1 mg ml^−1^ PEI and incubated for 10 min at room temperature. Six hundred microlitres of complete DMEM was added to the transfection mixture and this was immediately added to 4×10^5^ cells (washed twice with PBS). After 2 h at 37 °C, 5% CO_2_, cells were washed twice with PBS and the medium was replaced. For luciferase assays cells were harvested at 24 h p.t.

FHK cells were transduced with a lentivirus construct expressing pre-miR122 [61], and then selected with puromycin until a stable polyclonal population was obtained.

### *In vitro* transcription of RNA

Linearized DNA (2 μg) was used as a template in the T7 RiboMAX Large Scale RNA Production System (Promega). Reactions were incubated at 37 °C for 60 min before the degradation of template DNA using 2 units of DNase for 30 min at 37 °C. *In vitro*-transcribed (IVT) RNA was purified by acidic phenol/chloroform extraction and precipitated with isopropanol.

### RNA electroporation

For translation assays cells were harvested by centrifugation following trypsin treatment and washed twice with ice-cold PBS. Cells were counted and a final suspension of 5×10^6^ cells ml^−1^ was obtained in ice-cold DEPC-treated PBS. Four hundred microlitres of cell suspension was mixed with 2 μg RNA in a chilled electroporation cuvette (Geneflow) and cells were electroporated at 950 μF, 260 V for 25 ms (BioRad Gene Pulser). Cells were immediately recovered in complete media, seeded into culture plates and incubated at 37 °C, 5% CO_2_ until they were lysed for assay.

### FF luciferase assays

Plates seeded with cells following either electroporation (96-well plates, 2×10^5^ cells well^−1^) or transfection (6-well plates, 4×10^5^ cells well^−1^) were harvested for luciferase activity by washing in PBS and lysis in Passive Lysis Buffer (PLB; Promega), with 30 μl well^−1^ and 100 μl well^−1^, respectively. For SGR translation assays cells were harvested at 6 h p.t.; this provided the highest signals for replication-incompetent SGR (data not shown). For bicistronic translation assays cells were harvested 24 h p.t. Thirty microlitres of sample was transferred to a 96-well plate before 50 μl of Luciferase Assay Reagent (Promega) was added per well. Light emission was monitored on a BMG plate reader.

### SHAPE

Full-length *in vitro*-transcribed NZCI-luc RNA (12 pmol) was resuspended in 20 μl 0.5× TE buffer, incubated at 95 °C for 2 min and cooled on ice for 2 min. Following this, 103 μl ddH_2_O, 45 μl 3.3× folding buffer (333 mM HEPES, 20 mM MgCl_2_, 330 mM NaCl) and 2 μl RNAse inhibitor (RNAse UT, Invitrogen) was added and incubated for 30 min at 37 °C. After incubation the mixtures were evenly split into positive and negative reactions to which 8 μl of 100 mM NMIA (positive) or DMSO (negative) was added. Mixtures were incubated for 50 min at 37 °C and precipitated with 4 μl 5 M NaCl, 2 μl 100 mM EDTA, 1 μl 20 mg ml^−1^ glycogen, 18 μl ddH_2_O and 350 μl 100 % ethanol at −80 °C for 30 min. RNA was pelleted by centrifugation at 20000 ***g*** for 30 min at 4 °C, aspirated, dried and resuspended in 10 μl 0.5× TE buffer.

For both the positive and negative reactions 5 μl of this RNA was incubated with 1 μl 10 μM 5′ FAM-labelled fluorescent primer (5′ GTTCCATCCTCCAGAGGATAGAAT 3′, HPLC-purified) and 6 μl ddH_2_O at 85 °C for 1 min, 60 °C for 10 min and 30 °C for 10 min. A master mix of 4 μl superscript IV (SSIV) RT buffer, 1 μl 100 mM DTT, 0.5 μl 100 mM dNTPs, 0.5 μl RNAseOUT, 1 μl ddH_2_O and 1 μl SSIV RT was added to each reaction and incubated for 30 min at 55 °C. The primer was designed to bind in the luciferase gene, approximately 100 nt downstream of the AUG start codon.

For the sequencing ladder reaction 6 pmol of unfolded IVT RNA in 7.5 μl 0.5× TE buffer, 1 μl 10 mM 5′ HEX-labelled primer (HPLC purified) and 2 μl ddH_2_O was incubated at 85 °C for 1 min, 60 °C for 10 min and 30 °C for 10 min. A master mix of 4 μl SSIV RT buffer, 1 μl 100 mM DTT, 0.5 μl 100 mM dNTPs, 0.5 μl RNAseOUT, 2 μl ddGTP and 1 μl SSIV RT was added before incubation for 30 min at 55 °C. RT products were treated with 1M NaOH at 95 °C for 3 min and cooled on ice with 2 μl 2 M HCl for 2 min. cDNA was precipitated in 4 μl 3 M NaAc, 4 μl 100 mM EDTA, 1 μl 20 mg ml^−1^ glycogen and 60 μl 100% ethanol for 30 min at −80 °C, pelleted by centrifugation, aspirated and resuspended in 40 μl deionized formamide. Samples were pooled with 20 μl of ladder and stored at −80 °C prior to analysis.

For SHAPE footprinting, the 40S ribosomal subunit and purified initiation factor eIF3 were prepared from HeLa cells following established procedures [39, 62]. Following folding of the IVT RNA, 300 nM eIF3 or 40S ribosomal subunit was added and incubated for 20 min at 37 °C. NMIA or DMSO treatment was then conducted as described above.

### SHAPE data analysis

Capillary electrophoresis of SHAPE fragments was conducted by DNA Sequencing and Services (part of the MRC-PPU Reagents and Services Facility, College of Life Sciences, University of Dundee, UK). SHAPE data were analysed in the program QuSHAPE [63] using mostly default parameters, with the exception that the reactivity baseline was manually set to zero. RNA structure prediction was carried out using the RNAstructure software [64] using the SHAPE reactivity profile as a pseudo-free energy constraint. RNA secondary structure was modelled in VaRNA [65].

## Supplementary Material

Supplementary material

## Figures and Tables

**Table 1:** Sequence diversity in the apical loop SLIIIb across selected EHcV isolates

Isolate (GenBank accession)	Sequence (first nucleotide)
NZPI (NC_038425)	(244)GAACGUC..UUUGACC
JPN3 (AB863589)	(246)GAACGUCUGUAUGACC
SMKL2012 (JX948117)	(243)GAACGUCUGUAUGACC
Stewart *et al.* [8]	(243)GAACGUCACUUUGACC
H628 (MH028007)	(244)GAACGUC.UUAGGACC

## Abbreviations

EHcV: equine hepacivirus
HCV: hepatitis C virus
IRES: internal ribosome entry site
NMIA: *N*-methyl isatoic anhydride
SGR: sub-genomic replicon
SHAPE: selective 2′-hydroxyl acylation analyzed by primer extension
SL: stem loop
UTR: untranslated region
