# Supplementary material for: Structure–function analysis of the equine hepacivirus 5′ untranslated region highlights the conservation of translational mechanisms across the hepaciviruses
Source: J Gen Virol. Author manuscript; Available in PMC 2024 Mar 4. (PMC7615701; doi:10.1099/jgv.0.001316)
Supplement: Supplementary material [file EMS194314-supplement-Supplementary_material.pdf]

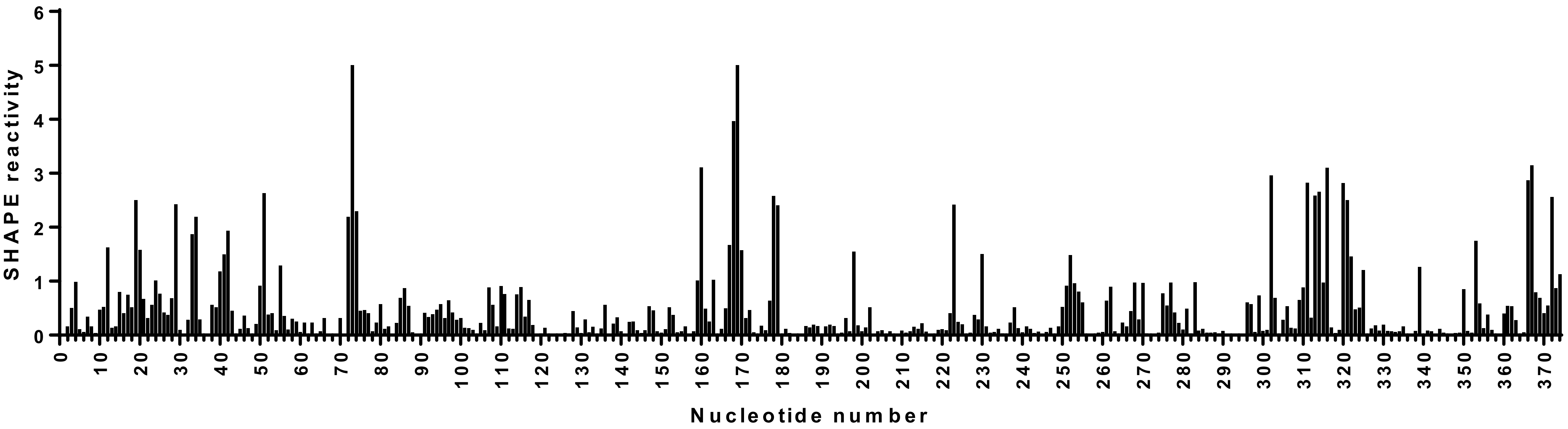

Supplementary Figure S1. Bar graph of SHAPE data for the complete EHcV 5′UTR. Values represent the average of two independent SHAPE reactions using the complete 5′UTR and correspond to the structure presented in Fig 2.

A

Nucleotide	WT no eIF3	WT +eIF3	Difference	p value
250 A	1.033	0.173	(0.860)	0.020
251 C	1.563	0.050	(1.513)	0.005
252 U	2.027	0.257	(1.770)	0.027
253 U	2.227	0.070	(2.157)	0.009
254 U	2.693	0.223	(2.470)	0.015

B

Nucleotide	WT no eIF3	GUC +eIF3	Difference	p value
250 A	1.033	0.790	(0.243)	0.173
251 C/G	1.563	1.807	0.240	0.224
252 U	2.027	2.367	0.340	0.401
253 U/C	2.227	0.770	(1.457)	0.015
254 U	2.693	2.873	0.180	0.293

C

Nucleotide	WT no 40S	WT +40S	Difference	p value
307 G	0.553	0.000	(0.553)	0.183
308 U	0.540	0.117	(0.423)	0.046
309 U	0.620	0.000	(0.620)	0.011
310 G	1.577	0.253	(1.323)	0.009
311 G	1.580	0.100	(1.480)	0.025
312 G	3.590	0.027	(3.563)	0.045
313 C	1.097	0.103	(0.993)	0.001
314 C	0.137	1.020	0.880	0.010

D

Nucleotide	WT no 40S	AGU +40S	Difference	p value
307 G	0.553	0.000	(0.553)	0.069
308 U	0.540	3.670	(3.130)	0.288
309 U	0.620	3.240	2.620	0.041
310 G/A	1.577	2.190	0.613	0.070
311 G	1.580	1.050	(0.530)	0.185
312 G/U	3.590	0.960	(2.630)	0.019
313 C	1.097	2.110	1.013	0.402
314 C	0.137	0.110	(0.027)	0.074

Supplementary Table S1: Numerical SHAPE data for the indicated residues (250-254 SLIIIb) and (307-314 SLIIId) +/- either eIF3 or 40S ribosomal subunit. (A) comparison of WT SLIIIb +/- eIF3. (B) comparison of WT SLIIIb alone with GUC substitution SLIIIb + eIF3. (C) comparison of WT SLIIId +/- 40S ribosomal subunit. (D) comparison of WT SLIIId alone with AGU substitution SLIIId + 40S ribosomal subunit. P values refer to difference from wildtype in the absence of eIF3 or 40S.

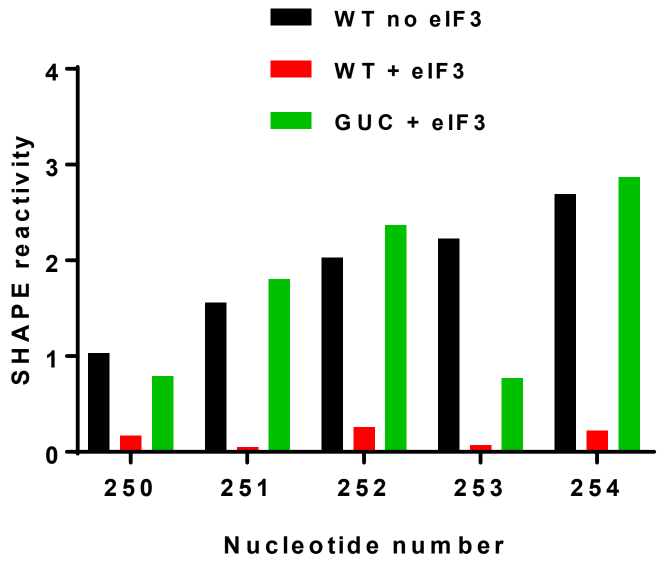

Supplementary Figure S2: Bar graph of numerical SHAPE data listed in Supplementary Table S1 A, B, and shown structurally in Fig 6 A-C, for the indicated residues (250-254 SLIIIb) +/- eIF3.

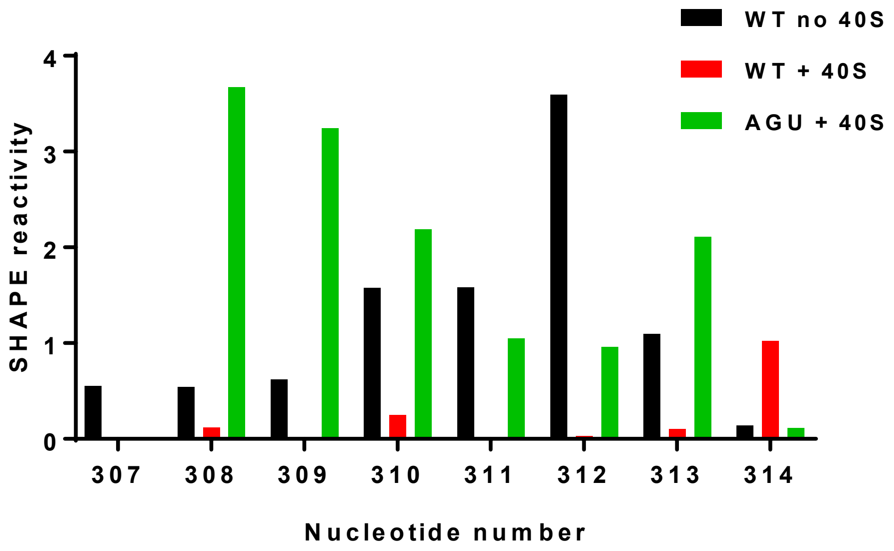

Supplementary Figure S3: Bar graph of numerical SHAPE data listed in Supplementary Table S1 C, D, and shown structurally in Fig 6 D-F, for the indicated residues (307-314 SLIIId) +/- 40S ribosomal subunit.

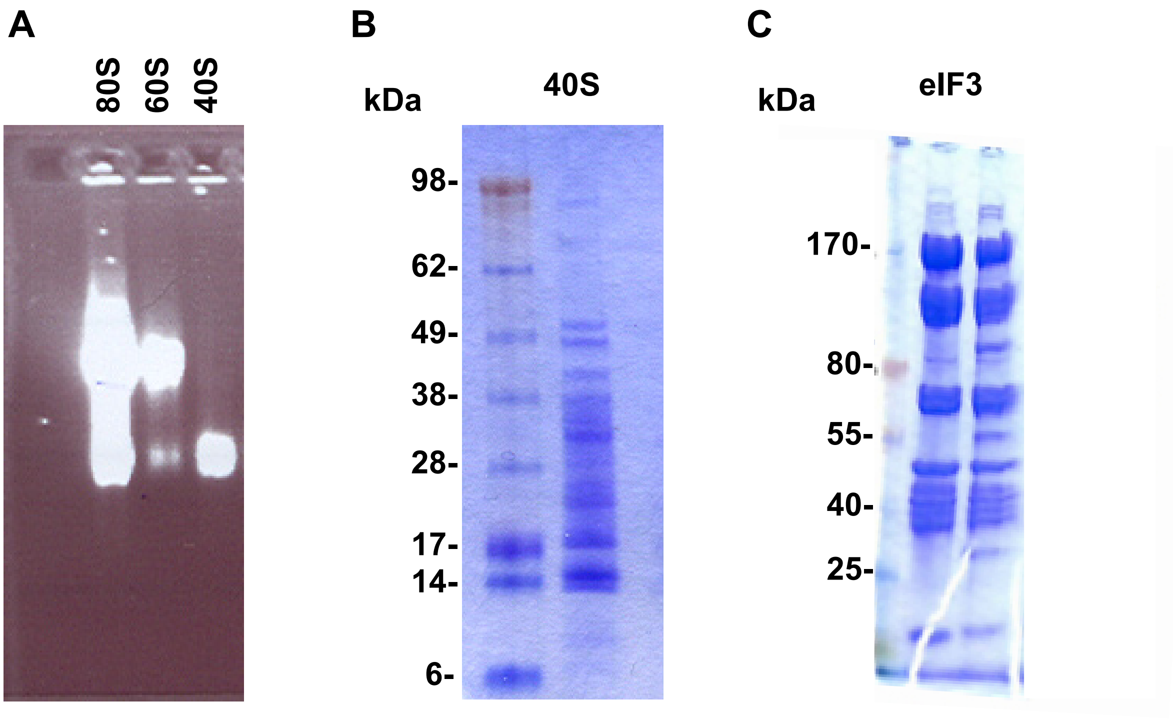

Supplementary Figure S4. Purity of eIF3 and 40S preparations. HeLa cytoplasmic extracts were processed as described previously (Pisarev AV *et al*, Methods in Enzymology (2007) 430:147-177). **A.** Agarose gel analysis of salt-washed ribosomes before puromycin treatment (80S), and following puromycin treatment and sucrose gradient fractionation of 40S and 60S. **B.** 40S ribosomal proteins were then fractionated on 4-20% NuPAGE gels. **C.** SDS-PAGE analysis of purified eIF3 from ribosome salt wash fractions following ammonium sulphate fractionation, MonoS and MonoQ Sepharose separation.
